# Supplementary material for: Cryo-EM structure of the mature and infective Mayaro virus at 4.4 Å resolution reveals features of arthritogenic alphaviruses
Source: Nat Commun. 2021 May 24;12:3038. doi: 10.1038/s41467-021-23400-9 (PMC8144435; doi:10.1038/s41467-021-23400-9)
Supplement: Supplementary file 2 — Supplementary Information [file 41467_2021_23400_MOESM2_ESM.pdf]

Cryo-EM structure of the mature and infective Mayaro virus at 4.4 Å resolution reveals features of arthritogenic alphaviruses

SUPPLEMENTARY INFORMATION

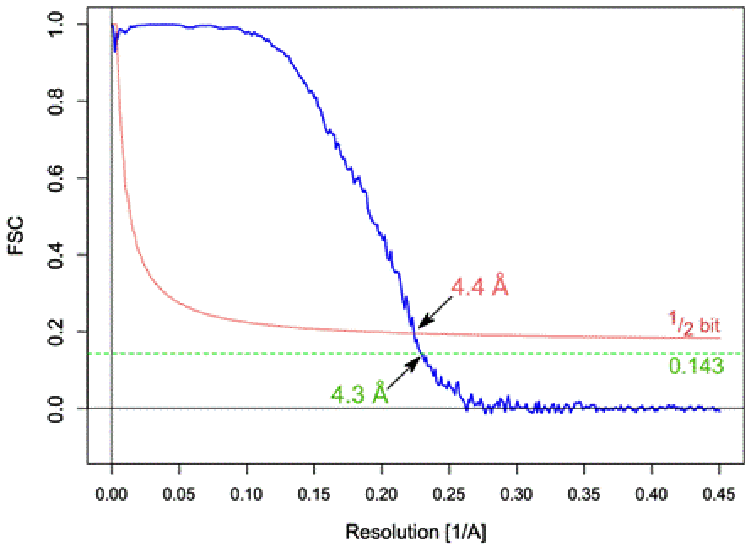

Supplementary Fig. 1. Fourier shell correlation profile of MAYV Cryo-EM density map. FSC curve calculated from two MAYV 3D volumes is shown in blue,the ½--bit curve in red and the 0.143 crossing line in green. The global resolution determined based on the ½--bit or 0.143 criterion is indicated60.

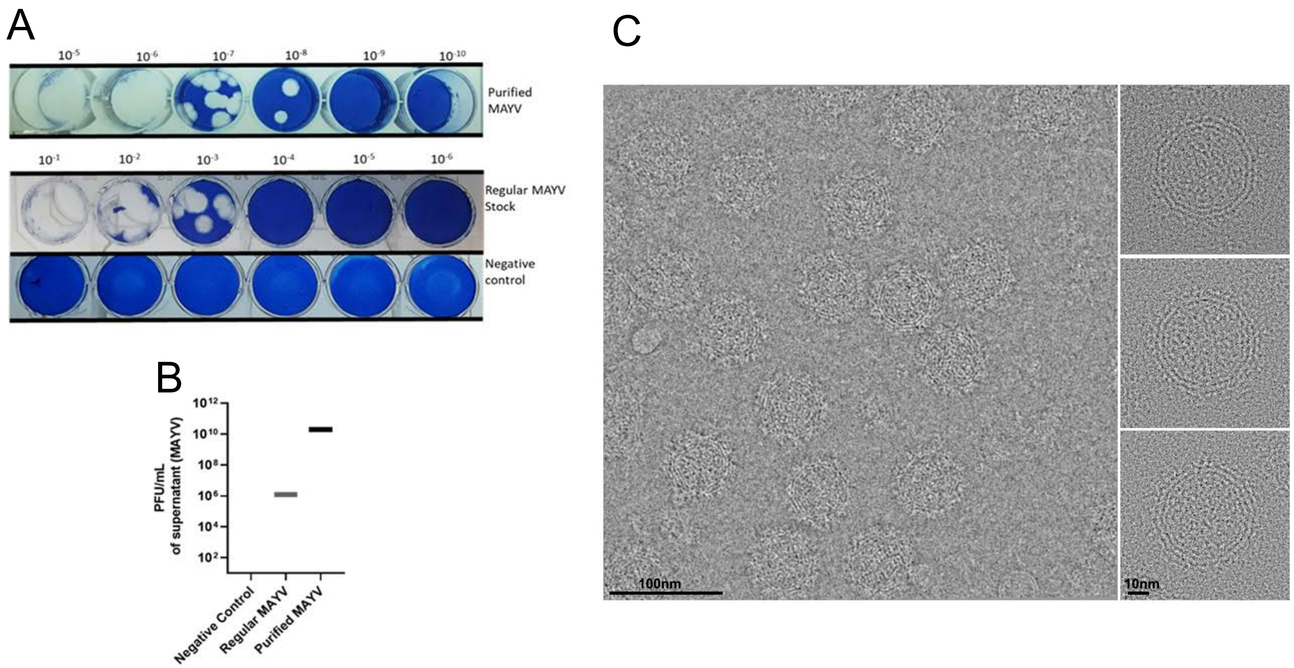

Supplementary Fig. 2. Purified MAYV stocks used in Cryo-EM experiments are viable and infective. (A) Representative pictures of plaque assays performed in Vero CCL81 cells to assess infective MAYV load in stock samples. Viable Vero CCL81 cells were stained in Methylene Blue 1% w/v. MAYV replication leads to cell death, creating transparent lysis plaques in the blue Vero CCL81 monolayer. **(B)** Viral load of stocks used in Cryo-EM experiments, before and after the purification process. (**C)** An example of MAYV Cryo-EM micrograph from set of 8792 movie stacks. The micrograph was processed by applying a band-pass filter in Imagic software to suppress very low-frequency information (less than 0.02 of Nyquist frequency), removing ramps of background fluctuations, and to suppress very high-frequency information (higher than 0.4 of Nyquist frequency), allowing a better visualization of low frequency content. After filtering, we resized the micrograph to 512x512 dimensions. Inset presents individually raw picked particles used in 3D reconstruction.

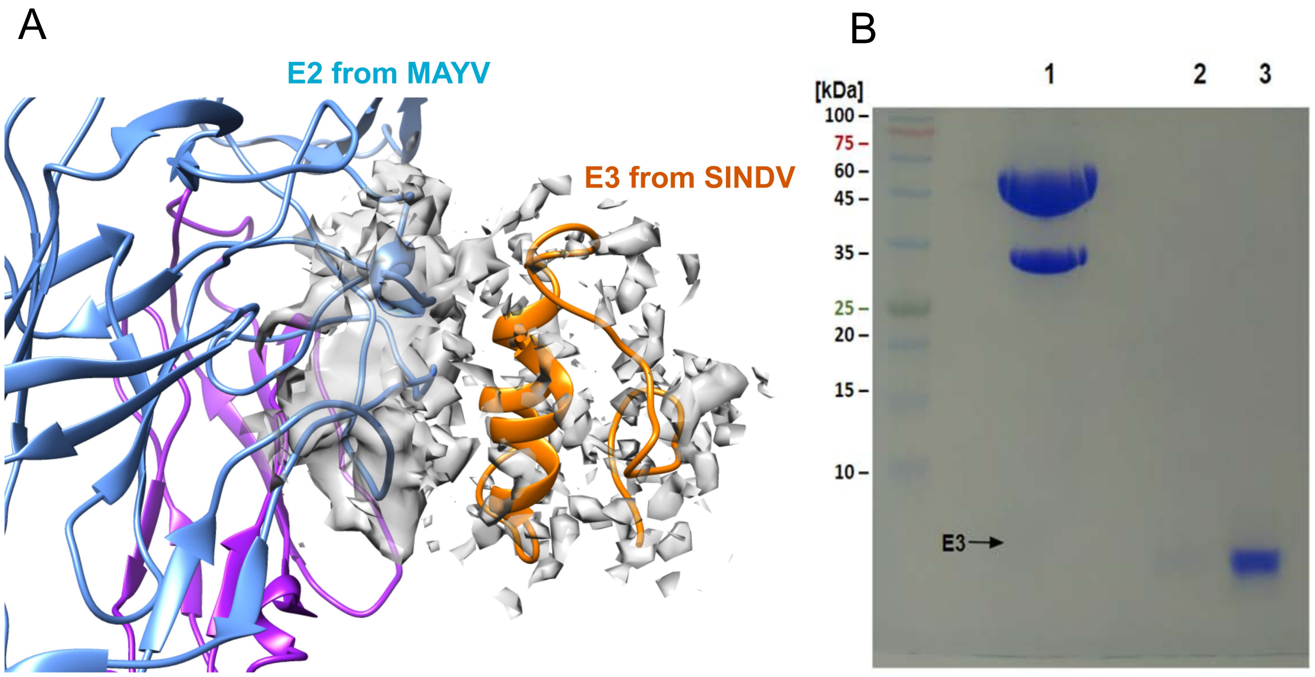

Supplementary Fig. 3. Absence of the E3 protein indicates that purified MAYV samples are composed of mature virions. (A) Superposition of the MAYV electron density with the SINDV particle cryo-EM structure (entry 6IMM). The SIDNV E3 protein is shown in orange. MAYV electron density is shown in grey contoured at 2.0 sigma-level. **(B)** Analysis of purified MAYV in 15% SDS-PAGE gel, stained with Coomassie brilliant blue. Lane 1 is carried with 30 µg of denatured purified MAYV and shows no visible bands compatible with E3 protein size (7 kDa). Lanes 2 and 3 are carried with 1 µg and 9 µg of a 5 kDa synthetic peptide, used as a positive control. This gel is representative of 7 experiments with similar results.

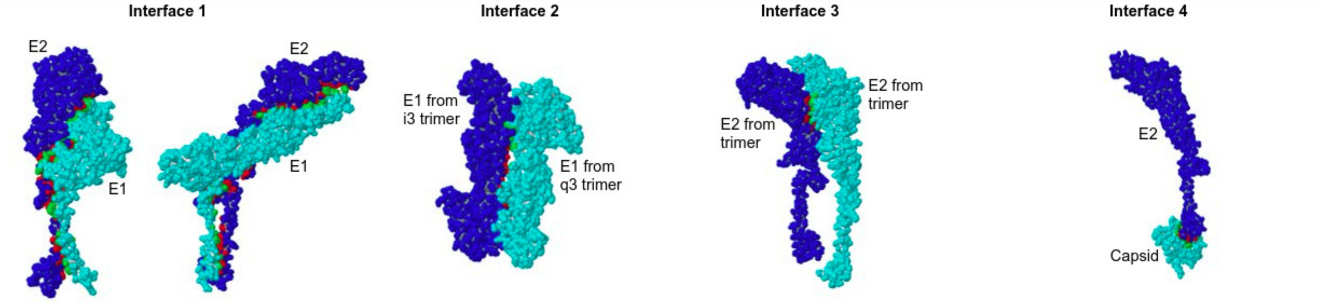

Averaged interface area (Å²)	N_{hb}	N_{sb}
2815	17	0

Averaged interface area (Å²)	N_{hb}	N_{sb}
554	1	1

Averaged interface area (Å²)	N_{hb}	N_{sb}
514	3	0

Averaged interface area (Å²)	N_{hb}	N_{sb}
541	2	0

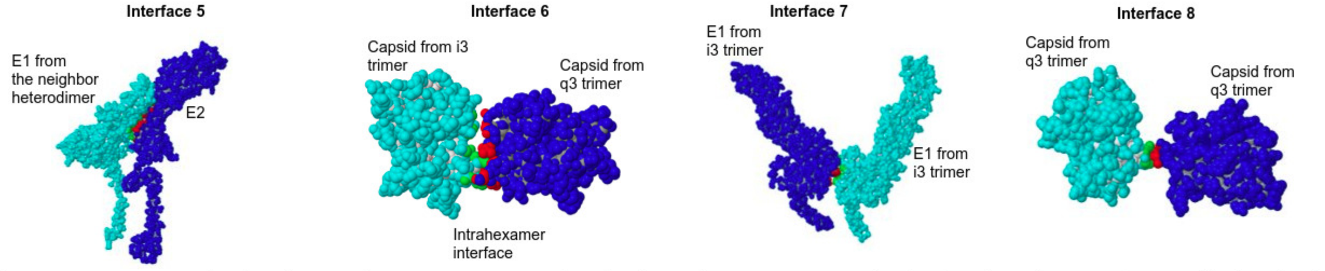

Averaged interface area (Å²)	N_{hb}	N_{sb}
463	2	1

Averaged interface area (Å²)	N_{hb}	N_{sb}
261	3	4

Averaged interface area (Å²)	N_{hb}	N_{sb}
254	2	0

Averaged interface area (Å²)	N_{hb}	N_{sb}
130	0	3

Supplementary Fig. 4. List of the main interfaces among MAYV structural proteins of the asymmetric unit. The interfaces were identified using the PDBePISA server (https://www.ebi.ac.uk/pdbe/prot_int/pistart.html). Only interfaces with area larger than 100 Å were considered. The structures are rendered as spacefill and the interaction partners are colored in blue or cyan. Interaction residues are colored in red or green. Nhb and Nsb represent the average number of hydrogen bonds and salt-bridges in each interface, respectively.

A - Multiple Sequence Alignment of the Alphaviral Capsid

```
      1        10        20          30              40
MAYV  MDFLPTQVFYGRRWR.PRM.PPR..PW....RPRPPTI....QRPDQQARQMQQLIAAVS
CHIKV MEFIPTQTFYNRRYQ.PRPWTPR..PTIQVIRPRPR........PQRQAGQLAQLISAVN
SFV   MNYIPTQTFYGRRWR.PRP.AAR..PWPLQATPVAPVV......PDFQAQQMQQLISAVN
RRV   MNYIPTQTFYGRRWR.PRP.AFR..PWQVSMQPTPTMVTPMLQAPDLQAQQMQQLISAVS
SINDV MN....RGFFNMLGRRPFPAPTA..MW....RPRRRRQAAPMPARNGLASQIQQLTTAVS
VEEV  FPFQPMYPMQPMPYRNPFAAPRR..PWFPRTDP.............FLAMQVQELTRSMA
EEEV  LNYPPMAPINPMAYRDPNP.PRQVAPF....RP.............PLAAQIEDLRRSIA

      50        60        70              80        90
MAYV  TLALRQ.NAAAPQRGRKKQPRRKKPKPQP......EKPKKQEQKPKQKKTP.........
CHIKV KLTMRA....VPQQKPRRNRKNKKQKQKQ......QAPQNNTNQKKQPPKKKPAQ.....
SFV   ALTMRQ.NAIAPARPPKPKKKKTTKPKPK......TQPKKINGKTQQQKKKDKQAD....
RRV   ALTTKQ.NVKAP.KGQRQKKQQKPKEKKE......NQKKKPTQKKKQQQKPKPQA.....
SINDV ALVIGQATRPQPPRPRPPPRQKKQAPKQP......PKPKKPKTQEKKKKQP.........
VEEV  NLTFKQ.RRDAPPEGPSAKKPKKEASQKQKGGGQGKKKKNQGKKKAKTGPPNPKAQNGNK
EEEV  NLTLKQ.RAPNPPAGPPAKRKK.............PAPSLSLETKKKRPPPPAKKQ....

              100       110        120       130       140
MAYV  ...KKKPGRRERMCMKIEHDCIFEVKHE.GKVTGYACLVGDKVMKPAHVPGVIDNIDLAR
CHIKV ..KKKKPGRRERMCMKIENDCIFEVKHE.GKVTGYACLVGDKVMKPAHVKGTIDNADLAK
SFV   .KKKKKPGKRERMCMKIENDCIFEVKHE.GKVTGYACLVGDKVMKPAHVKGVIDNADLAK
RRV   ..KKKKPGRRERMCMKIENDCIFEVKLD.GKVTGYACLVGDKVMKPAHVKGTIDNPDLAK
SINDV ..AKPKPGKRQRMALKLEADRLFDVKNEDGDVIGHALAMEGKVMKPLHVKGTIDHPVLSK
VEEV  KKTNKKPGKRQRMVMKLESDKTFPIMLE.GKINGYACVVGGKLFRPMHVEGKIDNDVLAA
EEEV  .KRKPKPGKRQRMCMKLESDKTFPIMLN.GQVNGYACVVGGRVFKPLHVEGRIDNEQLAA

     150        160       170       180       190       200
MAYV  LSYKKSSKYDLECAQIPVAMKSDASKYTHEKPEGHYNWHYGAVQYTGGRFTVPTGVGKPG
CHIKV LAFKRSSKYDLECAQIPVHMKSDASKFTHEKPEGYYNWHHGAVQYSGGRFTIPTGAGKPG
SFV   LAFKKSSKYDLECAQIPVHMRSDASKYTHEKPEGHYNWHHGAVQYSGGRFTIPTGAGKPG
RRV   LTYKKSSKYDLECAQIPVHMKSDASKYTHEKPEGHYNWHHGAVQYSGGRFTIPTGAGKPG
SINDV LKFTKSSAYDMEFAQLPVNMRSEAFTYTSEHPEGFYNWHHGAVQYSGGRFTIPRGVGGRG
VEEV  LKTKKASKYDLEYADVPQNMRADTFKYTHEKPQGYYSWHHGAVQYENGRFTVPKGVGAKG
EEEV  IKLKKASIYDLEYGDVPQCMKSDTLQYTSDKPPGFYNWHHGAVQYENNRFTVPRGVGGKG

     210        220       230       240       250
MAYV  DSGRPIFDNKGRVVAIVLGGANEGARTALSVVTWN.KDMVTKITPEGTEEW
CHIKV DSGRPIFDNKGRVVAIVLGGANEGARTALSVVTWN.KDIVTKITPEGAEEW
SFV   DSGRPIFDNKGRVVAIVLGGANEGSRTALSVVTWN.KDMVTRVTPEGSEEW
RRV   DSGRPIFDNKGRVVAIVLGGANEGARTALSVVTWT.KDMVTRVTPEGTEEW
SINDV DSGRPIMDNSGRVVAIVLGGADEGTRTALSVVTWNSKGKTIKTTPEGTEEW
VEEV  DSGRPILDNQGRVVAIVLGGVNEGSRTALSVVMWNEKGVTVKYTPENCEQW
EEEV  DSGRPILDNKGRVVAIVQGGVNEGSRTALSVVTWNQKGVTVKDTPEGSEPW
```

B - Multiple Sequence Alignment of the Alphaviral E1

```
      1        10        20        30        40        50        60        70        80
MAYV  YEHTAVIPNQVGFPYKAHVAREGYSPLTLQMQVVETSLEPTLNLEYITCDYKTKVPSPYVKCCGTAECRTQDKPEYKCAV
CHIKV YEHVTVIPNTVGVPYKTLVNRPGYSPMVLEMELLSVTLEPTLSLDYITCEYKTVIPSPYVKCCGTAECKDKNLPDYSCKV
SFV   YEHSTVMPNVVGFPYKAHIERPGYSPLTLQMQVVETSLEPTLNLEYITCEYKTVVPSPYVKCCGASECSTKEKPDYQCKV
RRV   YEHTATIPNVVGFPYKAHIERNGFSPMTLQLEVVETSWEPTLNLEYITCEYKTVVPSPFIKCCGTSECSSKEQPDYQCKV
SINDV YEHATTVPNVPQIPYKALVERAGYAPLNLEITVMSSEVLPSTNQEYITCKFTTVVPSPKIKCCGSLECQPAAHADYTCKV
VEEV  YEHATTMPSQAGISYNTIVNRAGYAPLPISITPTKIKLIPTVNLEYVTCHYKTGMDSPAIKCCGSQECTPTYRPDEQCKV
EEEV  YEHTAVMPNKVGIPYKALVERPGYAPVHLQIQLVNTRIIPSTNLEYITCKYKTKVPSPVVKCCGATQCTSKPHPDYQCQV

               90       100       110       120       130       140       150
MAYV  FTGVYPFMWGGAYCFCDSENTQMSEAYVERADVCKHDYAAAYRAHTASLRAKIKVTYGTVN.QTVEAYVNGDHAVTIAGT
CHIKV FTGVYPFMWGGAYCFCDAENTQLSEAHVEKSESCKTEFASAYRAHTASASAKLRVLYQGNN.ITVTAYANGDHAVTVKDA
SFV   YTGVYPFMWGGAYCFCDSENTQLSEAYVDRSDVCRHDHASAYKAHTASLKAKVRVMYGNVN.QTVDVYVNGDHAVTIGGT
RRV   YTGVYPFMWGGAYCFCDSENTQLSEAYVDRSDVCKHDHASAYKAHTASLKATIRISYGTIN.QTTEAFVNGEHAVNVGGS
SINDV FGGVYPFMWGGAQCFCDSENSQMSEAYVELSADCASDHAQAIKVHTAAMKVGLRIVYGNTT.SFLDVYVNGVTPGTSKDL
VEEV  FTGVYPFMWGGAYCFCDTENTQVSKAYVMKSDDCLADHAEAYKAHTASVQAFLNITVGEHS.IVTTVYVNGETPVNFNGV
EEEV  FTGVYPFMWGGAYCFCDTENTQMSEAYVERSEECSIDHAKAYKVHTGTVQAMVNITYGSVTWRSADVYVNGETPAKIGDA

    160       170       180       190       200       210       220       230
MAYV  KFIFGPVSTAWTPFDTKIVVYKGEVYNQDFPPYGAGQPGRFGDIQSRTLDSKDLYANTGLKLARPAAGNIHVPYTQTPSG
CHIKV KFIVGPMSSAWTPFDNKIVVYKGDVYNMDYPPFGAGRPGQFGDIQSRTPESKDVYANTQLVLQRPAAGTVHVPYSQAPSG
SFV   QFIFGPLSSAWTPFDNKIVVYKDEVFNQDFPPYGSGQPGRFGDIQSRTVESNDLYANTALKLARPSPGMVHVPYTQTPSG
RRV   KFIFGPISTAWSPFDNKIVVYKDDVYNQDFPPYGSGQPGRFGDIQSRTVESKDLYANTALKLSRPSPGVVHVPYTPTPSG
SINDV KVIAGPISASFTPFDHKVVIHRGLVYNYDFPEYGAMKPGAFGDIQATSLTSKDLIASTDIRLLKPSAKNVHVPYTQASSG
VEEV  KITAGPLSTAWTPFDRKIVQYAGEIYNYDFPEYGAGQPGAFGDIQSRTVSSSDLYANTNLVLQRPKAGAIHVPYTQAPSG
EEEV  KLIIGPLSSAWSPFDNKVVVYGHEVYNYDFPEYGTGKAGSFGDLQSRTSTSNDLYANTNLKLQRPQAGIVHTPFTQAPSG

    240       250       260       270       280       290       300       310
MAYV  FKTWQKDRDSPLNAKAPFGCTIQTNPVRAMNCAVGNIPVSMDIADSAFTRLTDAPIISELLCTVSTCTHSSDFGGVAVLS
CHIKV FKYWLKERGASLQHTAPFGCQIATNPVRAMNCAVGNMPISIDIPDAAFTRVVDAPSLTDMSCEVPACTHSSDFGGVAIIK
SFV   FKYWLKEKGTALNTKAPFGCQIKTNPVRAMNCAVGNIPVSMNLPDSAFTRIVEAPTIIDLTCTVATCTHSSDFGGVLTLT
RRV   FKYWLKEKGSSLNTKAPFGCKIKTNPVRAMDCAVGSIPVSMDIPDSAFTRVVDAPAVTDLSCQVVVCTHSSDFGGVATLS
SINDV FEMWKNNSGRPLQETAPFGCKIAVNPLRAVDCSYGNIPISIDIPNAAFIRTSDAPLVSTVKCEVSECTYSADFGGMATLQ
VEEV  FEQWKKDKAPSLKFTAPFGCEIYTNPIRAENCAVGSIPLAFDIPDALFTRVSETPTLSAAECTLNECVYSSDFGGIATVK
EEEV  FERWKRDKGAPLNDVAPFGCSIALEPLRPENCAVGSIPISIDIPDAAFTRISETPTVSDLECKITECTYASDFGGIATLP

    320       330       340       350       360       370       380       390
MAYV  YKVEKAGRCDVHSHSNVAVLQE..VSIEAEGRSVIHFSTASAAPSFIVSVCSSRATCTAKCEPPKDHVVTYPANHNGITL
CHIKV YAVSKKGKCAVHSMTNAVTIREAEIEVEGNSQLQISFSTALASAEFRVQVCSTQVHCAAECHPPKDHIVNYPASHTTLGV
SFV   YKTNKNGDCSVHSHSNVATLQEATAKVKTAGKVTLHFSTASASPSFVVSLCSARATCSASCEPPKDHIVPYAASHSNVVF
RRV   YKTDKPGKCAVHSHSNVATLQEATVDVKEDGKVTVHFSTASASPAFKVSVCDAKTTCTAACEPPKDHIVPYGASHNNQVF
SINDV YVSDREGQCPVHSHSSTATLQESTVHVLEKGAVTVHFSTASPQANFIVSLCGKKTTCNAECKPPADHIVSTPHKNDQEFQ
VEEV  YSASKSGKCAVHVPSGTATLKEAAVELTEQGSATIHFSTANIHPEFRLQICTSYVTCKGDCHPPKDHIVTHPQYHAQTFT
EEEV  TNPVKQETVQFIVHQVLQLLKRMTSPLLRAGSFTFHFSTANIHPAFKLQVCTSGITCKGDCKPPKDHIVDYPAQHTESFT

      400       410       420       430
MAYV  PDLSSTAMTWAQHLAGGVGLLIALAVLILVIVTC..ITLRR..
CHIKV QDISATAMSWVQKITGGVGLVVAVAALILIVVLC..VSFSRH.
SFV   PDMSGTALSWVQKISGGLGAFAIGAILVLVVVTC..IGLRR..
RRV   PDMSGTAMTWVQRLASGLGGLALIAVVVLVLVTC..ITMRR..
SINDV AAISKTSWSWLFALFGGASSLLIIGLMIFACSMM..LTSTRR.
VEEV  AAVSKTAWTWLTSLLGGSAVIIIIGLVLATIVAMYVLTNQKHN
EEEV  SAISATAWSWLKVLVGGTSAFIVLGLIATAVVAL.VLFFHRH.
```

C - Multiple Sequence Alignment of the Alphaviral E2

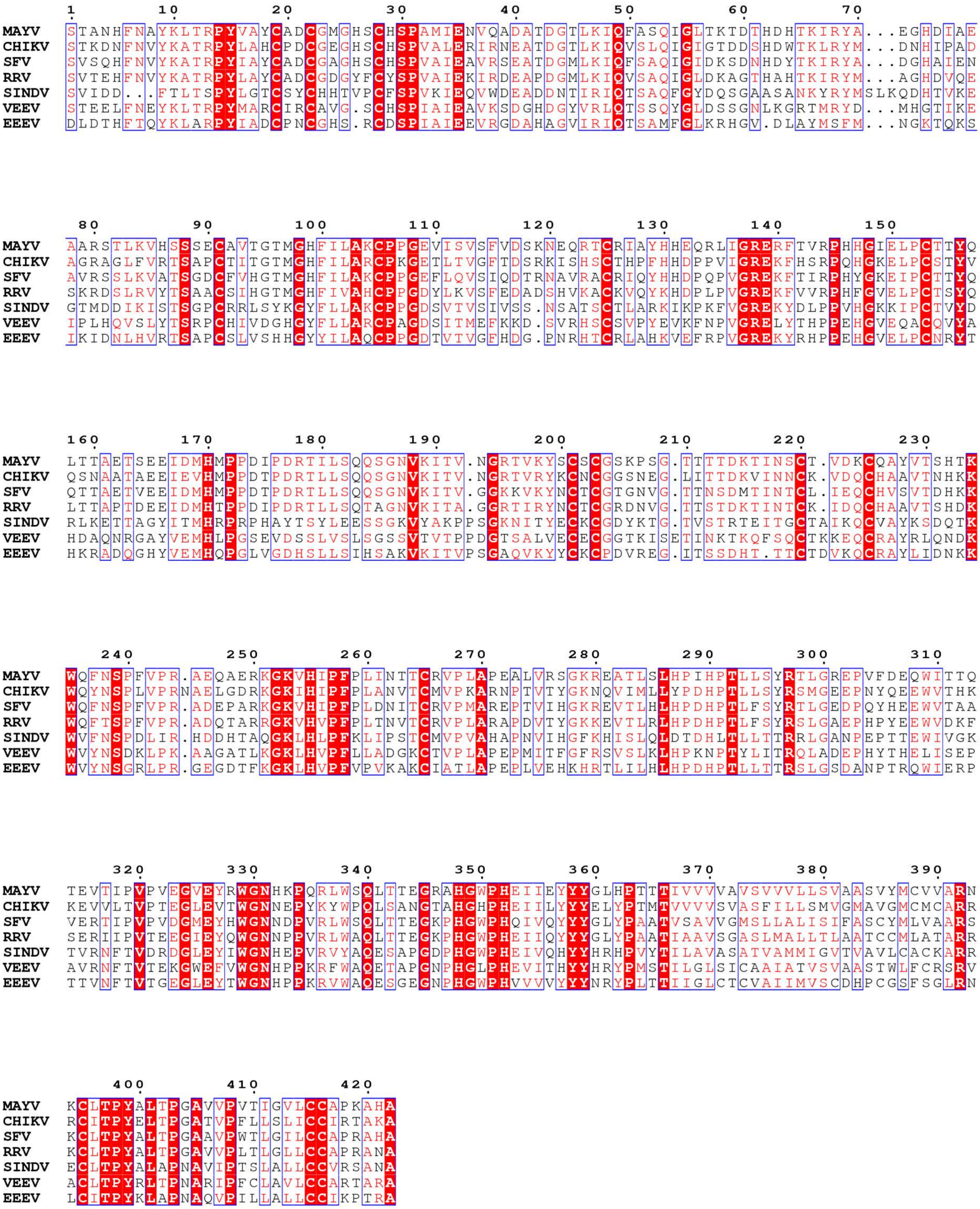

Supplementary Fig. 5. Multiple sequence alignment of alphaviruses structural proteins. The alignments were performed for proteins C **(A)**, E1 **(B)** and E2 **(C)** using the Muscle Algorithm in EBI server (https://www.ebi.ac.uk/Tools/msa/muscle/) and visualized with ESPript version 3.0 server.

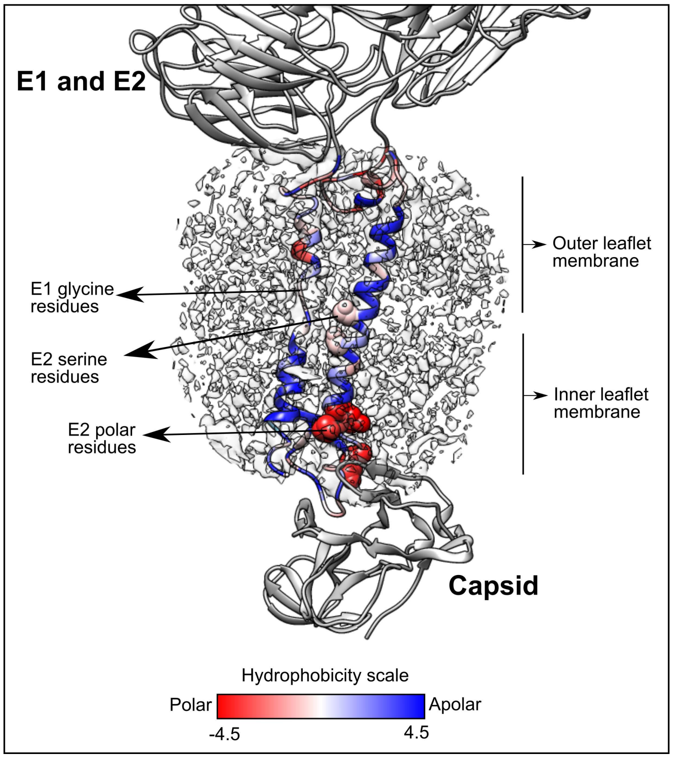

Supplementary Fig. 6. MAYV E1 and E2 transmembrane domains. Overall view of E1 and E2 TM domains inserted into the lipid membrane. The 3D atomic model is colored by hydrophobicity using Kyte-Doolittle scale. Important arginine and lysine residues in E2 are highlighted in spheres, as well as glycine (E1) and serine (E2) residues. The electron density is shown in grey surface.

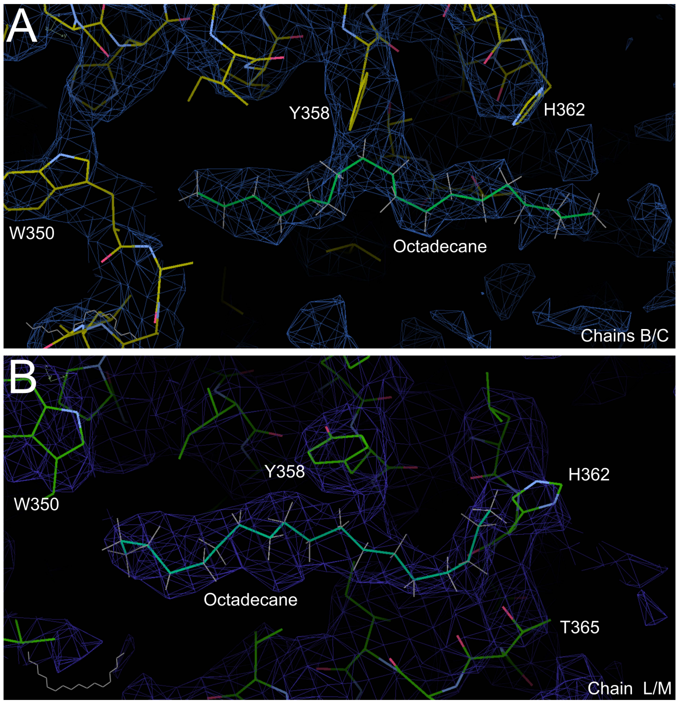

Supplementary Fig. 7. 3D model fitting of a C18 hydrocarbon (Octadecane) in the extra density from two MAYV E1-E2 heterodimers (chains B/C and chains L/M). Octadecane was built and fitted into the density map using Coot. The density map was rendered at 2.5 sigma contour level.

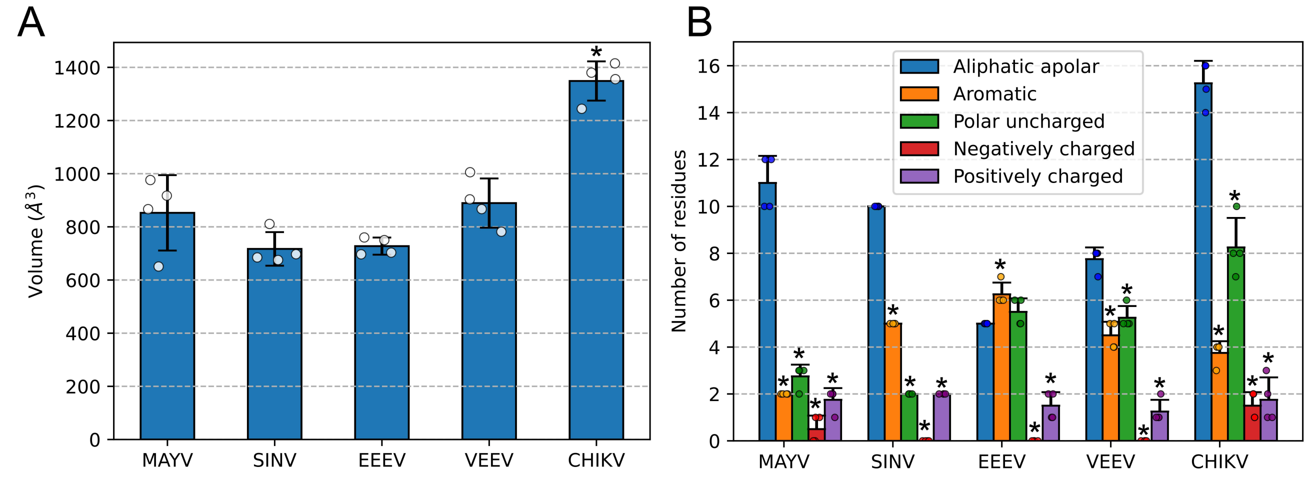

Supplementary Fig. 8. Structural features extracted from the cavity between E1 and E2 TM helices in MAYV and other alphaviruses. (A) Cavity volume estimated for the four E1-E2 heterodimers (n = 4 independent heterodimer structures) in asymmetric unit. One-way ANOVA with Tukey's multiple comparison test was used for comparing MAYV cavity volume with other alphaviruses (* indicates adj. p < 0.01 when comparing the alphaviruses to MAYV). **(B)** Number of residues in each four E1-E2 heterodimers (n = 4 independent heterodimer structures) in asymmetric unit separated by classes. One-way ANOVA with Tukey's multiple comparison test was used for comparing the number of residues in aliphatic apolar class with the other classes in the same alphavirus species (* indicates adj. p < 0.01 when comparing the aliphatic apolar class with the other classes). All data are presented as mean values +/- SD. Aliphatic apolar: ALA, VAL, ILE, LEU, GLY, PRO; Aromatic: PHE, TYR, TRP; Polar uncharged: SER, THR, CYS, MET, ASN, GLN; Negatively charged: GLU, ASP; Positively charged: ARG, LYS, HIS.

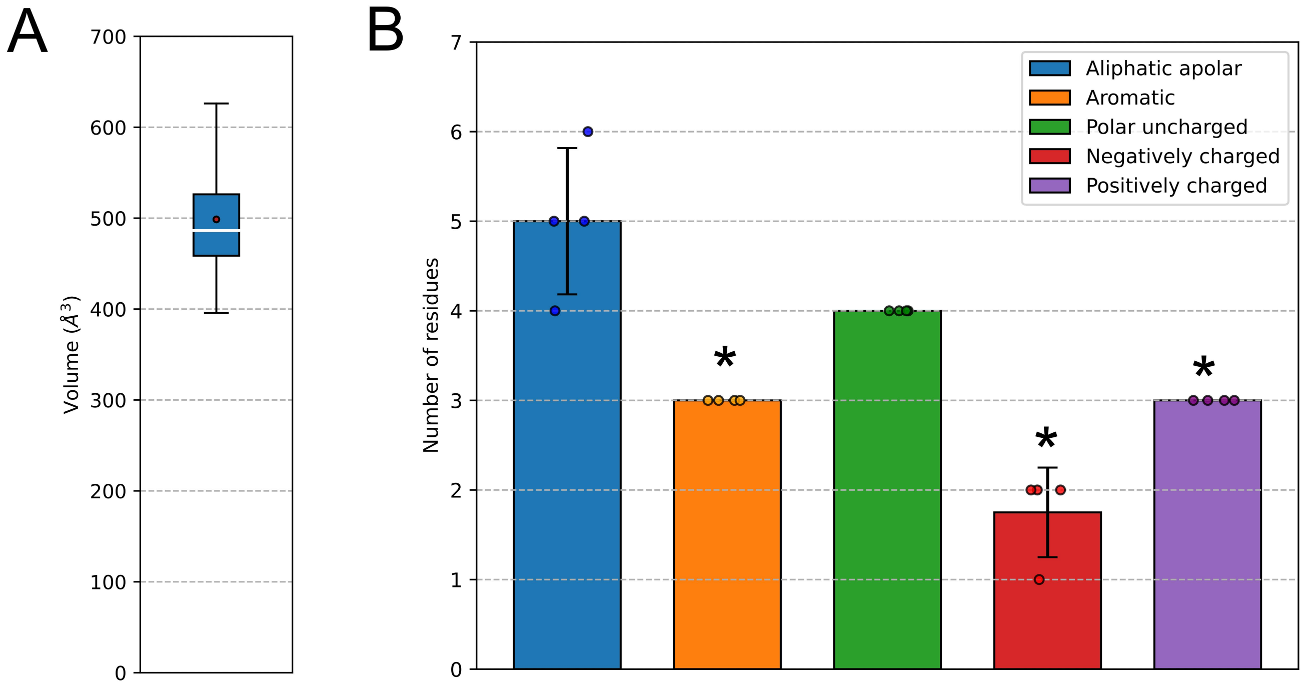

Supplementary Fig. 9. Structural features extracted from the C-protein cavity that binds to E2 C-terminal. (A) Boxplot of cavity volume estimated for the four capsids (n = 4 independent capsids structures) in asymmetric unit. In the boxplot, the box portion defines the interquartile range (IQR) (67.5 Å3) and the 75th (Q3) (526.2 Å3) and 25th (Q1) (458.7 Å3) percentiles. The central line indicates the median (486.3 Å3) and the mean (498.6 Å3) is indicated by a dot. The whiskers with minimum (395.7 Å3) and maximum (626.2 Å3) are determined using Q1-1.5 x IQR and Q3+1.5 x IQR, respectively. **(B)** Number of residues in each four capsids (n = 4 independent capsids structures) in asymmetric unit separated by classes. One-way ANOVA with Tukey's multiple comparison test was used for comparing the number of residues in aliphatic apolar class with the other classes (* indicates adj. p < 0.01 when comparing the aliphatic apolar class with the other classes) All data are presented as mean values +/- SD. Aliphatic apolar: ALA, VAL, ILE, LEU, GLY, PRO; Aromatic: PHE, TYR, TRP; Polar uncharged: SER, THR, CYS, MET, ASN, GLN; Negatively charged: GLU, ASP; Positively charged: ARG, LYS, HIS.

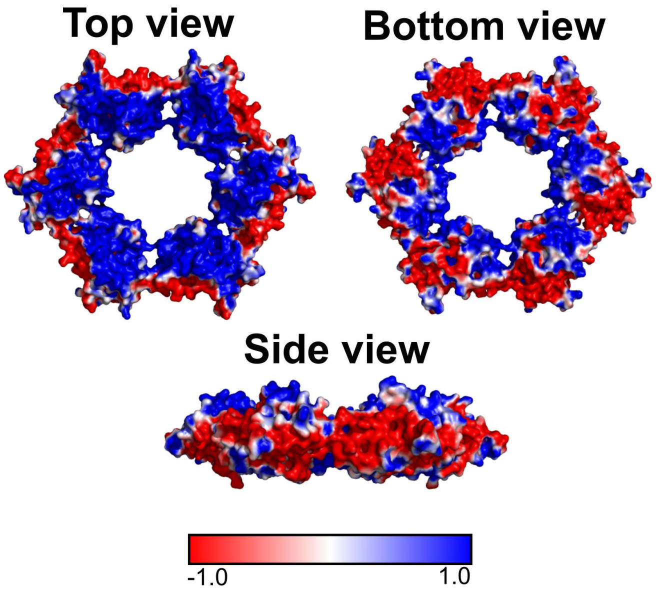

Supplementary Fig. 10. The electrostatic potential of at the surface of MAYV capsid proteins. The electrostatic potential was calculated using ABPS and visualized in Pymol. Top, bottom and side view from capsid hexameric organization is shown. Charges are presented in a gradient of blue (positive) to red (negative), white being neutral local charge.

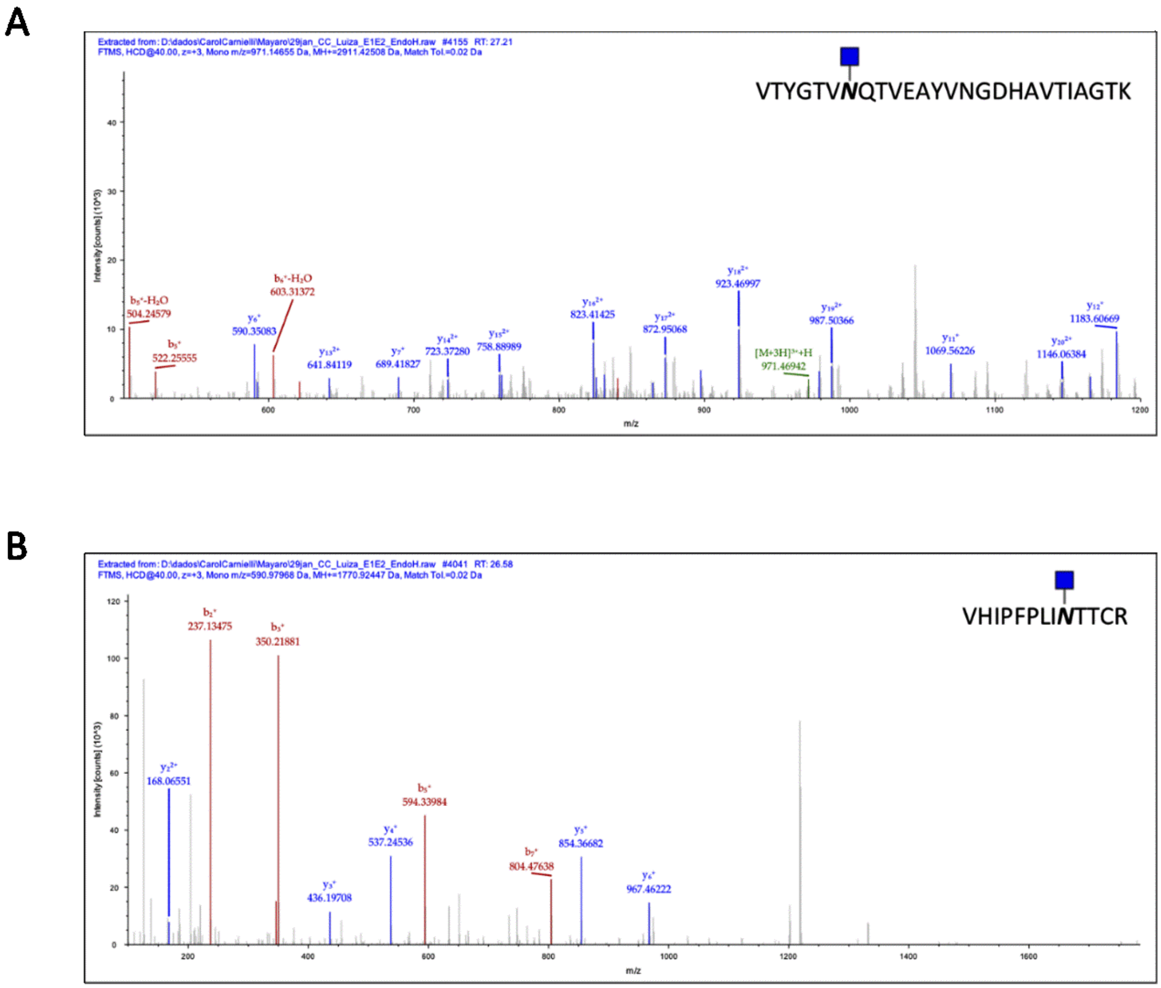

Supplementary Fig. 11. Spectra of identified peptides, after Endo H and trypsin digestions, annotated by Proteome Discoverer and manually verified. (A) Spectrum of the peptide VTYGTVNQTVEAYVNGDHAVTIAGTK (m/z 971.14655, +3) from E1 protein with the N-acetylglucosamine in N141 residue, and **(B)** VHIPFPLINTTCR (m/z 590.97968, +3) E2 peptide with the N-acetylglucosamine in the N262 residue. The blue square represents the N-Acetyl-D-Glucosamine monosaccharide.

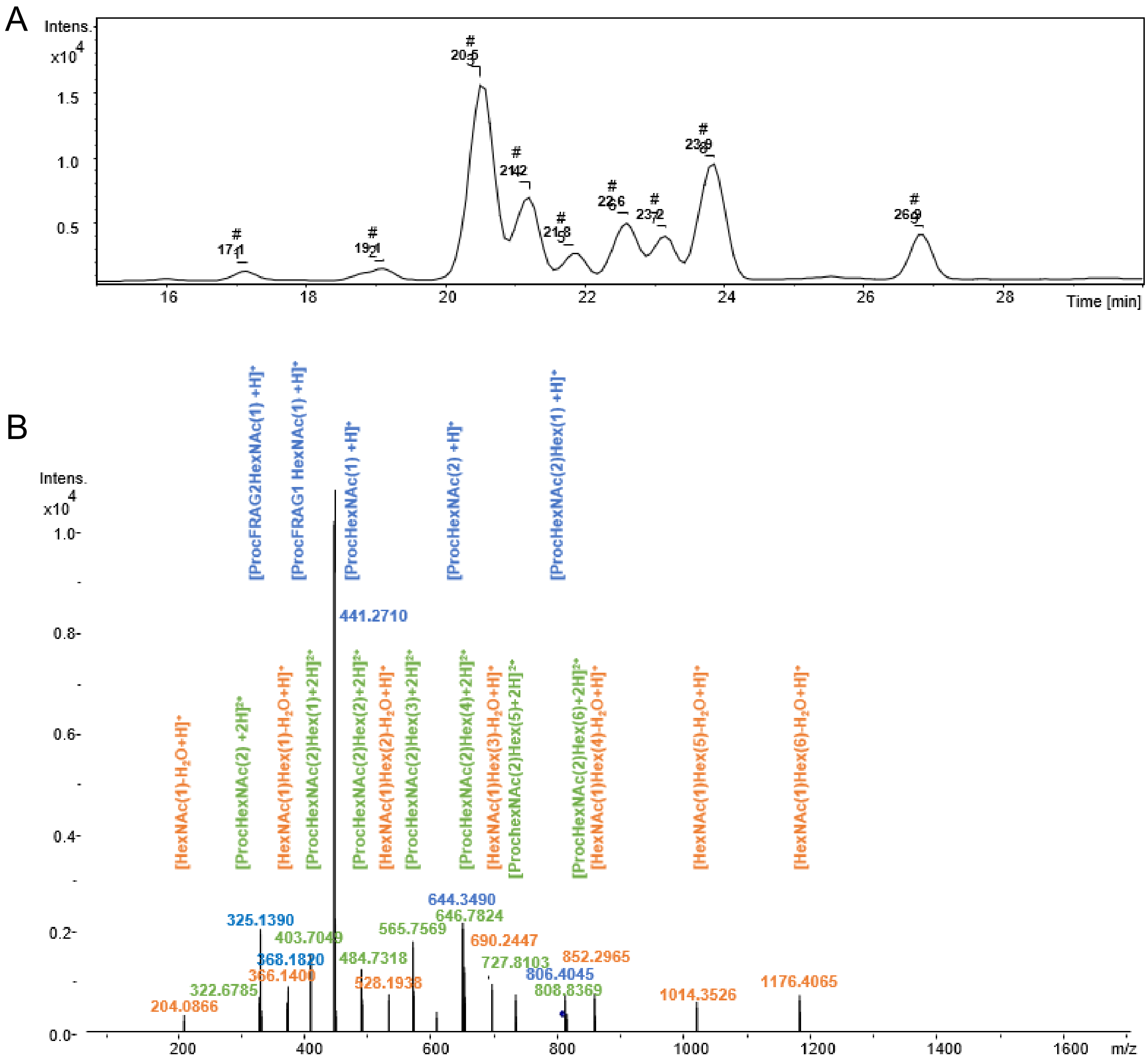

Supplementary Fig. 12. Base peak chromatogram derived from UPLC-MS/MS analyses of N-glycans released from E1/E2 glycoproteins of MAYV and glycan composition interpretation based on MS2 data. (A) Nine peaks were integrated (#1 to #9) and characterized as N-glycans composed by N-acetyl hexosamine (HexNAc); hexose (Hex) and fucose (Fuc) - HexNAc(2-5)Hex(5-8)Fuc(0-1). These glycan were proposed based on MS1 isotopic pattern (presence of double and triple charge forms in a variation of adducts) and respective carbohydrate unit compositions were proposed based on MS2 fragmentation pattern, as exemplified for peak #3 in (B). **(B)** MS2 (fragmentation) spectrum of m/z 808.8387, the most intense MS1 peak detected in the chromatographic peak #3. Through the interpretation of different MS1 peaks, according to the isotopic pattern for double and triple charge and also the variation of adducts (please see Supplementary Table 5 or details), the m/z 808.8387 was identified as [M+2H]2+, thus representing a glycan of 1,615.6597 Da composed by HexNAc(2)Hex(6). In the fragmentation spectrum, it was possible to recognize neutral losses of 162.05 (relative to hexose residues) and 203.07 (N-acetyl-hexosamine residues), in addition to fragments corresponding to procainamide derivatives (orange and blue sequence paths, respectively). Furthermore, MS2 double charge peaks fragmentation sequence (green) allowed for the visualization of losses of 81.02 (another way to verify the presence of the hexose units). For the other six chromatogram peaks (peaks #4 to #9), the same rational was used to investigate the fragmentation pattern and to attribute the respective compositions of the glycans **(Figure 4D)**. For peaks #1 and #2, it composition was predicted mainly using MS1 data, due to the poor fragmentation in the MS2 data. All N-glycans compositions were confirmed using Glycoworkbench software and data was deposited at UniCarb-DB.

Supplementary Table 1. Identity between structural proteins from MAYV and other alphaviruses.

	CHIKV strain S27	SFV	RRV strain T48	SINDV	EEEV strain Florida 91-469	VEEV strain TC-83
Capsid – disorder N-terminal (1-102)	53 %	56 %	63 %	43 %	33 %	37 %
Capsid - Structured domain (103-258)	89 %	90 %	90 %	65 %	63 %	64 %
E1	62 %	72 %	71 %	48 %	52 %	52 %
E2	56 %	67 %	62 %	37 %	41 %	38 %

Supplementary Table 2. Cryo-EM data collection and processing.

Magnification (x)	59,000
Voltage (kV)	300
Electron exposure (e- Å-2)	30
Nominal defocus (μm)	-1.5
Pixel size (Å)	1.1074
Symmetry imposed	Icosahedral
Number of micrographs	175840 (8792 movie stacks)
Initial number of particles images	79000
Number of particle images used in the final 3D reconstruction	40179
Map global resolution	4.4 (1⁄2-bit threshold) 4.3 (0.143 threshold)

Supplementary Table 3. Overall MAYV 3D atomic model quality evaluated by MolProbity.

**Structural composition**	
Chains	12
Residues	4004
**MolProbity statistics**	
Bonds length (Å) (RMSD - # > 4 sigmas)	0.006 (0)
Bond angles (°) (RMSD - # > 4 sigmas)	1.070 (43)
MolProbity score	2.0
Clash score	8.7
Ramachandran plot (%)	
Outliers	0.1
Allowed	11.4
Favored	88.6
Rotamer outliers (%)	0.59
Cβ outliers (%)	0
**Model vs. Data**	
CC (mask)	0.69

Supplementary Table 4. List of alphaviruses PDB files and Cryo-EM maps used for comparative studies.

Alphavirus	PDB ID	EMD ID	Method	Resolution	Reference
CHIKV	6NK5, 6NK6	9393	Cryo-EM	~4.5 A	Basore, 2019
SINDV (only E1 and E2)	6IMM	9693	Cryo-EM	~3.5 A	Chen, 2018
VEEV	3J0C	5275	Cryo-EM	~4.4 A	Zhang, 2011
EEEV	6MX4	9280	Cryo-EM	~4.4 A	Hasan, 2018

Supplementary Table 5. Summary of MS[1] analyses of N-glycans released from E1/E2 MAYV and identified by UPLC-MS/MS analyses. The nine chromatographic peaks are presented with featured MS[1] variations (double and triple charge and adducts). The glycan composition was proposed in combination with MS[2] data.

Peaks	RT (min)	Peak Area	$[M+2H]^{2+}$	$[M+HK]^{2+}$	$[M+2K]^{2+}$	$[M+3H]^{3+}$	$[M+2HK]^{3+}$	$[M+2KH]^{3+}$	$[M+3K]^{3+}$	M[a]	Proposed Composition[b]
#1	17.1	14739	727.8114	746.7888	ND	ND	ND	ND	ND	1453.6066	HexNAc(2)Hex(5)
#2	19.1	25597	829.3501	848.3246	ND	ND	565.8864	578.5398	ND	1656.6851	HexNAc(3)Hex(5)
#3	20.5	298547	808.8383	827.8154	846.7937	ND	552.213	564.8646	ND	1615.6597	HexNAc(2)Hex(6)
#4	21.2	78873	930.8921	949.8696	968.8477	620.9288	633.5809	646.2322	658.8851	1859.7648	HexNAc(4)Hex(5)
#5	21.8	25492	1032.4332	1051.4114	ND	688.622	701.2741	713.9259	726.5785	2062.8460	HexNAc(5)Hex(5)
#6	22.6	60892	1003.9234	1022.9008	1041.8813	669.6158	682.2672	694.9192	707.5719	2005.8260	HexNAc(4)Hex(5)Fuc(1)
#7	23.2	27692	1105.4638	1124.4417	ND	737.3091	749.9607	762.6121	775.2670	2208.9063	HexNAc(5)Hex(5)Fuc(1)
#8	23.9	195538	889.8666	908.844	927.822	ND	606.2306	618.8828	631.5339	1777.7148	HexNAc(2)Hex(7)
#9	26.9	76399	970.8955	989.8735	1008.8516	ND	660.2494	672.9011	685.5527	1939.7717	HexNAc(2)Hex(8)

[a]For N-glycans exact mass, it was considered the average of the most intense MS[1] peaks

[b]Derived from MS and MS[2] peak interpretation of N-glycans reacted with procainamide

ND - not detected
