# Supplementary material for: Cryo-EM structure of the mature and infective Mayaro virus at 4.4 Å resolution reveals features of arthritogenic alphaviruses
Source: Nat Commun. 2021 May 24;12:3038. doi: 10.1038/s41467-021-23400-9 (PMC8144435; doi:10.1038/s41467-021-23400-9)
Supplement: Supplementary file 3 — Description of Additional Supplementary Files [file 41467_2021_23400_MOESM3_ESM.pdf]

Description of Additional Supplementary Files

Liquid chromatography-Mass spectrometry/ Mass spectrometry (LC-MS/MS) data analysis of MAYV proteins indicate N-glycosylated sites at positions N141 in E1 and N262 in E2. Viral protein bands were separated in SDS-PAGE and digested with PNGaseF or EndoH. Digested proteins and a non-digested protein control were later digested with trypsin and analyzed at the mass spectrometer coupled to nanoflow liquid chromatography.

Supplementary Data 1 - List of protein groups identified in samples digested with trypsin.

Supplementary Data 2 - List of peptides identified in samples digested with trypsin.

Supplementary Data 3 - List of protein groups identified in samples digested with PNGase F and trypsin.

Supplementary Data 4 - List of peptides identified in samples digested with PNGase F and trypsin.

Supplementary Data 5 - List of protein groups identified in samples digested with Endo H and trypsin.

Supplementary Data 6 - List of peptides identified in samples digested with Endo H and trypsin.
