# Supplementary material for: Cryo-EM structure of the mature and infective Mayaro virus at 4.4 Å resolution reveals features of arthritogenic alphaviruses
Source: Nat Commun. 2021 May 24;12:3038. doi: 10.1038/s41467-021-23400-9 (PMC8144435; doi:10.1038/s41467-021-23400-9)
Supplement: Supplementary file 10 — Reporting summary [file 41467_2021_23400_MOESM10_ESM.pdf]

nature research

Corresponding author(s): Marques, R. E.

Last updated by author(s): Apr 26, 2021

Reporting Summary

Nature Research wishes to improve the reproducibility of the work that we publish. This form provides structure for consistency and transparency in reporting. For further information on Nature Research policies, see our Editorial Policies and the Editorial Policy Checklist.

Statistics

For all statistical analyses, confirm that the following items are present in the figure legend, table legend, main text, or Methods section.

n/a	Confirmed	
☐	☒	The exact sample size (*n*) for each experimental group/condition, given as a discrete number and unit of measurement
☐	☒	A statement on whether measurements were taken from distinct samples or whether the same sample was measured repeatedly
☐	☒	The statistical test(s) used AND whether they are one- or two-sided<br>*Only common tests should be described solely by name; describe more complex techniques in the Methods section.*
☐	☒	A description of all covariates tested
☐	☒	A description of any assumptions or corrections, such as tests of normality and adjustment for multiple comparisons
☐	☒	A full description of the statistical parameters including central tendency (e.g. means) or other basic estimates (e.g. regression coefficient) AND variation (e.g. standard deviation) or associated estimates of uncertainty (e.g. confidence intervals)
☐	☒	For null hypothesis testing, the test statistic (e.g. *F*, *t*, *r*) with confidence intervals, effect sizes, degrees of freedom and *P* value noted<br>*Give P values as exact values whenever suitable.*
☒	☐	For Bayesian analysis, information on the choice of priors and Markov chain Monte Carlo settings
☒	☐	For hierarchical and complex designs, identification of the appropriate level for tests and full reporting of outcomes
☒	☐	Estimates of effect sizes (e.g. Cohen's *d*, Pearson's *r*), indicating how they were calculated

Our web collection on statistics for biologists contains articles on many of the points above.

Software and code

Policy information about availability of computer code

Data collection	EPU software (version 2.5.0.4799REL) was used for data collection.
Data analysis	IMAGIC-4D (version July 2020) was used for image processing and single particle analysis. Local resolution of Cryo-EM density map was estimated using Bsoft (version 2.0.7). UCSF Chimera (version 1.13.1) was used for 3D atomic model and Cryo-EM density map visualization. parKVFinder (version 1.0) was used to prospect cavities in 3D atomic model.

For manuscripts utilizing custom algorithms or software that are central to the research but not yet described in published literature, software must be made available to editors and reviewers. We strongly encourage code deposition in a community repository (e.g. GitHub). See the Nature Research guidelines for submitting code & software for further information.

Data

Policy information about availability of data

All manuscripts must include a data availability statement. This statement should provide the following information, where applicable:

- Accession codes, unique identifiers, or web links for publicly available datasets
- A list of figures that have associated raw data
- A description of any restrictions on data availability

The cryoEM datasets generated and analysed during the current study are available in the EMDB repository, entry EMD-22961 [https://www.ebi.ac.uk/pdbe/entry/emdb/EMD-22961], and in the PDB repository, entry 7KO8 [https://www.rcsb.org/structure/7KO8]. The mass spectrometry-based proteomics data have been deposited to the ProteomeXchange Consortium via the PRIDE partner repository with the dataset identifier PXD024432 [http://proteomecentral.proteomexchange.org/cgi/GetDataset?ID=PXD024432]. The UPLC-MS/MS datasets are deposited at GlycoPOST under the accession code GPST000171 [https://glycopost.glycosmos.org/entry/GPST000171].MAYV strain IQT 4235 genome is deposited at GenBank under accession number MK070491.1.

April 2020

Field-specific reporting

Please select the one below that is the best fit for your research. If you are not sure, read the appropriate sections before making your selection.

☒ Life sciences ☐ Behavioural & social sciences ☐ Ecological, evolutionary & environmental sciences

For a reference copy of the document with all sections, see nature.com/documents/nr-reporting-summary-flat.pdf

Life sciences study design

All studies must disclose on these points even when the disclosure is negative.

Sample size	A total of 175840 raw micrographs (4096 x 4096) (8792 movie stacks) were used in data processing.
Data exclusions	Viral particles in raw micrographs that were clumped, defective or superimposed were excluded from analysis. Micrographs were pre-processed through a posteriori camera correction to minimize spurious correlations from camera imperfections.
Replication	A total of 175840 raw micrographs were collected at the microscope in 6 different occasions, originated from 4 different grid preparations and 3 different purified MAYV stocks, in a period of 3 years.
Randomization	Not applicable.
Blinding	Not applicable.

Reporting for specific materials, systems and methods

We require information from authors about some types of materials, experimental systems and methods used in many studies. Here, indicate whether each material, system or method listed is relevant to your study. If you are not sure if a list item applies to your research, read the appropriate section before selecting a response.

Materials & experimental systems

n/a	Involved in the study
☒	☐ Antibodies
☐	☒ Eukaryotic cell lines
☒	☐ Palaeontology and archaeology
☐	☒ Animals and other organisms
☒	☐ Human research participants
☒	☐ Clinical data
☒	☐ Dual use research of concern

Methods

n/a	Involved in the study
☒	☐ ChIP-seq
☒	☐ Flow cytometry
☒	☐ MRI-based neuroimaging

Eukaryotic cell lines

Policy information about cell lines

Cell line source(s)	Vero CCL81 cells were purchased from Banco de Células do Rio de Janeiro (BCRJ, Brazil).
Authentication	None of the cell lines used were authenticated by us.
Mycoplasma contamination	Vero CCL81 Cell line tested negative for mycoplasma contamination.
Commonly misidentified lines (See ICLAC register)	We used Vero CCL81 cells, not to be confused with Vero E6 cells.

Animals and other organisms

Policy information about studies involving animals; ARRIVE guidelines recommended for reporting animal research

Laboratory animals	The study did not involve laboratory animals
Wild animals	The study did not involve wild animals
Field-collected samples	The study did not involve samples collected from field
Ethics oversight	No ethical approval was required, because no animals were involved in the study.

Note that full information on the approval of the study protocol must also be provided in the manuscript.
